# Dietary amino acid intakes associated with a low-phenylalanine diet combined with amino acid medical foods and glycomacropeptide medical foods and neuropsychological outcomes in subjects with phenylketonuria

**DOI:** 10.1016/j.dib.2017.06.004

**Published:** 2017-06-07

**Authors:** Bridget M. Stroup, Sangita G. Murali, Nivedita Nair, Emily A. Sawin, Fran Rohr, Harvey L. Levy, Denise M. Ney

**Affiliations:** a Department of Nutritional Sciences, University of Wisconsin-Madison, WI, United States; b Division of Genetics and Genomics, Boston Children׳s Hospital, Harvard Medical School, Boston, MA, United States

**Keywords:** Tyrosine, Leucine, Arginine, Executive function, Delis-Kaplan Executive Function System, Cambridge Neuropsychological Test Automated Battery

## Abstract

This article provides original data on median dietary intake of 18 amino acids from amino acid medical foods, glycomacropeptide medical foods, and natural foods based on 3-day food records obtained from subjects with phenylketonuria who consumed low-phenylalanine diets in combination with amino acid medical foods and glycomacropeptide medical foods for 3 weeks each in a crossover design. The sample size of 30 subjects included 20 subjects with classical phenylketonuria and 10 with a milder or variant form of phenylketonuria. Results are presented for the Delis-Kaplan Executive Function System and the Cambridge Neuropsychological Test Automated Battery; the tests were administered at the end of each 3-week dietary treatment with amino acid medical foods and glycomacropeptide medical foods. The data are supplemental to our clinical trial, entitled “Glycomacropetide for nutritional management of phenylketonuria: a randomized, controlled, crossover trial, 2016 (1) and “Metabolomic changes demonstrate reduced bioavailability of tyrosine and altered metabolism of tryptophan via the kynurenine pathway with ingestion of medical foods in phenylketonuria, 2017 (2). This data has been made public and has utility to clinicians and researchers due to the following: 1) This provides the first comprehensive report of typical intakes of 18 amino acids from natural foods, as well as amino acid and glycomacropeptide medical foods in adolescents and adults with phenylketonuria; and 2) This is the first evidence of similar standardized neuropsychological testing data in adolescents and adults with early-treated phenylketonuria who consumed amino acid and glycomacropeptide medical foods.

**Specifications Table**

**Table:** 

Subject area	*Biology, Medicine*
More specific subject area	*Inherited Metabolic Disorders*
Type of data	*Figure (study design), Tables (dietary amino acid intakes, neuropsychological testing outcomes)*
How data was acquired	*Assessment of dietary intake of amino acids and neuropsychological function in patients with PKU*
Data format	*Analyzed data, mean±SD, median (25th–75th percentile)*
Experimental factors	*Data of subjects with PKU enrolled in Clinical trial at Waisman center, Madison, WI and Boston Children׳s hospital, Boston, MA.*
Experimental features	*Randomized Crossover Clinical Trial*
Data source location	*Madison, Wisconsin, USA*
Data accessibility	*The data are accessible within the article*

**Value of the data**

• The data presented are the first comparison of how ingestion of medical foods comprised primarily of single amino acids or intact protein from glycomacropeptide (a 64-amino acid glycophosphopeptide isolated from cheese whey) affect the dietary intake profile of amino acids.
• The dietary intake of 18 amino acids provides useful information to clinicians and researchers related to typical amino acid intake of individuals with phenylketonuria.
• The data from the standardized neuropsychological tests can be compared with pharmacological studies using these same tests to contrast the effectiveness of dietary management with pharmacological treatment in subjects with phenylketonuria [3], [4].
• These data are useful to clinicians and researchers evaluating the safety and efficacy of glycomacropeptide medical foods in the nutritional management of phenylketonuria.

## 1 Data

Summary data for dietary intake of amino acids and assessment of neuropsychological and executive function are presented for subjects with phenylketonuria enrolled in a randomized, controlled, crossover trial conducted from November 2010 to July 2015 [1], [2]. These data are herein reported for the first time (Table 1, Table 2, Table 3). The trial is registered at www.clinicaltrials.gov as NCT01428258 .

**Table 1:** Daily dietary intake profile of 18 amino acids of the low-phenylalanine diet in combination with amino acid medical foods and glycomacropeptide medical foods in participants with classical and variant phenylketonuria.

**Amino acids**	**Dietary treatment**^a^,b				**PKU Genotype**^e^				**p-values**		
	**AA-MF**^c^		**GMP-MF**^d^		**Classical**		**Variant**		**trt**	**gt**	**trt × gt**
**Alanine**											
g Ala/d	4.0	(2.9–5.0)	3.3	(2.6–3.5)	3.5	(2.9–4.3)	3.3	(2.7–4.0)	0.002	0.56	0.39
g Ala from MF/d	3.4	(2.0–4.0)	2.2	(1.7–2.8)	2.7	(2.0–3.7)	2.0	(1.6–2.9)	0.001	0.15	0.23
g Ala from NF/d	0.7	(0.6–1.1)	0.8	(0.6–1.3)	0.7	(0.5–1.1)	1.0	(0.7–1.7)	0.81	0.07	0.38
**Arginine**											
g Arg/d	5.1	(4.3–6.7)	6.0	(5.3–7.3)	6.0	(4.8–7.2)	5.6	(4.1–7.0)	0.01	0.59	0.45
g Arg from MF/d	4.2	(2.9–5.5)	4.9	(4.0–6.5)	4.9	(3.8–6.1)	4.1	(2.9–5.8)	0.01	0.31	0.30
g Arg from NF/d	0.9	(0.7–1.3)	0.9	(0.6–1.6)	0.8	(0.6–1.3)	1.1	(0.8–2.0)	0.93	0.13	0.52
**Aspartate**											
g Asp/d^f^	6.3	(5.2–7.4)	5.3	(4.7–6.0)	5.8	(5.0–7.1)	5.3	(4.2–6.5)	0.01	0.23	–
g Asp from MF/d	4.7	(2.8–5.9)	3.2	(2.4–4.0)	4.1	(2.9–5.3)	2.7	(2.1–3.8)	0.01	0.08	0.11
g Asp from NF/d	1.7	(1.4–2.9)	1.9	(1.3–2.7)	1.6	(1.3–2.6)	2.0	(1.6–3.7)	0.74	0.18	0.68
**Cysteine**											
Cys/d	3.0	(2.5–3.5)	0.6	(0.4–0.8)	1.5	(0.6–3.0)	1.5	(0.6–3.0)	<0.0001	0.52	0.12
g Cys from MF/d^f^	2.2	(1.9–3.0)	0.01	(0–0.03)	0.4	(0.01–2.4)	0.4	(0.02–2.1)	<0.0001	0.87	–
g Cys from NF/d	0.6	(0.4–0.8)	0.6	(0.4–0.8)	0.5	(0.4–0.7)	0.7	(0.5–1.3)	0.75	0.10	0.64
**Glutamate**											
g Glu/d	7.1	(4.2–13.5)	12.1	(9.9–15.0)	11.5	(7.5–14.7)	10.2	(5.9–12.7)	0.0003	0.64	0.10
g Glu from MF/d	0.6	(0–9.1)	7.2	(5.6–8.9)	7.2	(2.7–9.5)	3.9	(0–7.0)	0.0002	0.07	0.07
g Glu from NF/d	4.1	(2.9–5.5)	4.8	(3.0–6.2)	3.8	(2.7–5.5)	5.1	(3.1–8.2)	0.91	0.16	0.83
**Glycine**											
g Gly/d	4.0	(3.4–5.8)	1.1	(0.9–1.4)	2.3	(1.1–4.2)	2.8	(1.1–3.7)	<0.0001	0.74	0.51
g Gly from MF/d	3.3	(2.6–4.9)	0.4	(0.3–0.5)	1.0	(0.4–3.7)	1.0	(0.4–3.0)	<0.0001	0.44	0.57
g Gly from NF/d	0.6	(0.5–0.9)	0.7	(0.5–1.0)	0.6	(0.5–0.8)	0.8	(0.6–1.6)	0.82	0.11	0.28
**Histidine**											
g His/d	2.4	(2.1–2.7)	2.0	(1.5–2.1)	2.2	(1.8–2.6)	2.1	(1.6–2.4)	0.002	0.73	0.55
g His from MF/d	1.8	(1.5–2.3)	1.3	(1.0–1.7)	1.8	(1.3–2.1)	1.4	(1.0–1.7)	<0.0001	0.11	0.39
g His from NF/d	0.4	(0.3–0.6)	0.5	(0.3–0.7)	0.4	(0.3–0.6)	0.6	(0.4–2.0)	0.63	0.09	0.81
**Isoleucine**											
g Ile/d	4.1	(3.7–5.3)	5.0	(3.6–5.6)	4.8	(3.9–5.6)	4.0	(3.6–5.3)	0.09	0.26	0.17
g Ile from MF/d	3.4	(2.8–4.0)	3.8	(2.9–4.9)	3.8	(3.2–5.0)	2.9	(2.5–3.7)	0.16	0.08	0.15
g Ile from NF/d	0.7	(0.5–1.0)	0.8	(0.5–1.2)	0.6	(0.5–1.0)	1.1	(0.6–1.6)	0.61	0.07	0.77
**Leucine**											
g Leu/d	7.4	(6.4–9.2)	12.5	(9.9–14.8)	10.2	(8.0–13.0)	7.8	(6.5–12.1)	<0.0001	0.34	0.21
g Leu from MF/d	6.3	(4.4–7.5)	10.4	(8.7–13.3)	8.8	(6.3–10.9)	6.3	(4.8–9.6)	<0.0001	0.15	0.18
g Leu from NF/d	1.3	(1.0–1.8)	1.5	(0.9–2.7)	1.2	(0.9–1.8)	1.9	(1.1–3.0)	0.59	0.07	0.68
**Lysine**											
g Lys/d	5.5	(4.4–6.6)	3.2	(3.0–3.7)	4.0	(3.2–5.9)	4.1	(3.3–5.1)	<0.0001	0.84	0.05
g Lys from MF/d	4.8	(3.2–5.1)	2.2	(1.8–2.8)	3.0	(2.2–4.9)	2.6	(2.0–3.3)	<0.0001	0.06	0.01
g Lys from NF/d	0.7	(0.5–1.2)	0.9	(0.6–1.5)	0.7	(0.5–1.1)	1.3	(0.7–2.0)	0.68	0.049	0.94
**Methionine**											
g Met/d	1.5	(1.2–2.1)	1.2	(1.0–1.5)	1.4	(1.1–1.7)	1.3	(1.1–1.7)	0.004	0.60	0.34
g Met from MF/d	1.0	(0.8–1.6)	0.7	(0.5–1.0)	1.0	(0.8–1.3)	0.7	(0.5–0.9)	0.0001	0.04	0.07
g Met from NF/d	0.4	(0.2–0.5)	0.4	(0.2–0.7)	0.3	(0.2–0.5)	0.5	(0.3–0.9)	0.63	0.09	0.54
**Phenylalanine**											
mg Phe/d	924	(663-1,187)	1,014	(707-1,433)	855	(569-1,180)	1,082	(814-1,973)	0.25	0.06	0.75
mg Phe from MF/d	0	(0-0)	85	(70–110)	4	(0–92)	25	(0–80)	<0.0001	0.73	–
mg Phe from NF/d	924	(663-1,187)	929	(640-1,383)	797	(544-1,167)	1,065	(734-1,973)	0.97	0.07	0.92
**Proline**											
g Pro/d	5.9	(5.2–6.7)	6.3	(5.1–7.5)	6.3	(5.3–6.9)	5.5	(4.9–7.0)	0.28	0.85	0.25
g Pro from MF/d	4.3	(3.3–5.9)	4.5	(3.6–5.7)	4.6	(3.9–5.8)	3.9	(3.1–4.6)	0.88	0.20	0.26
g Pro from NF/d	1.2	(0.8–1.9)	1.6	(0.9–2.2)	1.1	(0.7–1.8)	1.8	(0.9–2.5)	0.36	0.12	0.91
**Serine**											
g Ser/d	3.7	(3.1–4.7)	3.8	(3.0–4.2)	3.8	(3.2–4.5)	3.5	(3.0–4.3)	0.28	0.59	0.86
g Ser from MF/d	2.7	(2.0–3.5)	2.5	(1.8–3.1)	2.9	(2.1–3.6)	2.2	(1.7–2.7)	0.13	0.15	0.74
g Ser from NF/d	0.8	(0.6–1.1)	0.9	(0.5–1.3)	0.7	(0.5–1.1)	1.1	(0.8–1.8)	0.72	0.10	0.79
**Threonine**											
g Thr/d	3.5	(3.1–4.4)	7.0	(5.3–8.0)	5.0	(3.6–7.0)	4.3	(3.4–6.5)	<0.0001	0.37	0.53
g Thr from MF/d	3.0	(2.2–3.3)	5.8	(4.6–7.3)	4.1	(3.0–6.0)	3.2	(2.2–5.2)	<0.0001	0.18	0.45
g Thr from NF/d	0.6	(0.5–0.9)	0.7	(0.5–1.1)	0.6	(0.5–0.9)	0.9	(0.6–1.4)	0.68	0.10	0.55
**Tryptophan**											
g Trp/d^f^	1.4	(1.2–1.8)	1.1	(0.9–1.2)	1.2	(1.0–1.5)	1.2	(1.0–1.3)	0.0006	0.35	–
g Trp from MF/d	1.2	(0.9–1.4)	0.8	(0.6–0.9)	1.0	(0.8–1.3)	0.8	(0.6–1.0)	<0.0001	0.10	0.03
g Trp from NF/d	0.2	(0.2–0.3)	0.2	(0.1–0.4)	0.2	(0.1–0.3)	0.3	(0.2–0.5)	0.81	0.22	0.87
**Tyrosine**											
g Tyr/d	6.1	(5.1–7.8)	5.2	(3.4–6.2)	6.0	(4.5–7.7)	5.1	(3.9–6.1)	0.0004	0.21	0.07
g Tyr from MF/d	5.6	(4.0–7.3)	3.8	(2.3–5.2)	5.3	(3.8–6.4)	3.9	(3.1–5.0)	0.0003	0.11	0.08
g Tyr from NF/d^f^	0.7	(0.4–1.0)	0.6	(0.4–1.2)	0.6	(0.4–0.8)	0.9	(0.5–1.4)	0.81	0.02	–
**Valine**											
g Val/d	4.9	(4.3–6.2)	4.6	(3.5–5.0)	4.8	(4.2–5.9)	4.5	(4.0–5.0)	0.03	0.29	0.07
g Val from MF/d	4.1	(3.1–4.8)	3.3	(2.6–4.2)	4.1	(3.1–4.8)	3.0	(2.6–4.0)	0.002	0.07	0.04
g Val from NF/d	0.9	(0.6–1.3)	1.0	(0.7–1.4)	0.8	(0.6–1.3)	1.2	(0.8–2.0)	0.68	0.08	0.71

AA-MF, amino acid medical food; GMP-MF, glycomacropeptide medical food; MF, medical foods; NF, natural foods; PKU, phenylketonuria.

Values are medians with the 25th to 75th percentile values in parentheses, based on consecutive 3-d food records at the end of the 3-wk AA-MF and GMP-MF treatments, *n*=30. Statistical analysis included ANOVA with effects for treatment (trt, AA-MF or GMP-MF), genotype (gt, Classical or Variant PKU), and treatment by genotype interaction (trt × gt). When data was skewed, the Kruskal-Wallis test was used.

a Medical food was defined as any medical food intended for the treatment of PKU.

b Natural food was defined as any food or beverage that was not intended for the treatment of PKU.

c The AA-MFs consumed during the study (2010–2015) included the following: CAMINO PRO PKU, Lophlex Power, Periflex Advance, Periflex Junior, Phenex-2, PhenylAde Essential Drink Mix, PhenylAde MTE Amino Acid Blend, Phenyl-Free 2, Phenyl-Free 2 HP, Phlexy-10 Drink Mix, Phlexy-10 Tablets, PKU Cooler 15, PKU Cooler 20, PKU Lophlex LQ 20, and XPhe Maxamum Powder.

d The Cambrooke Therapeutics GMP-MFs consumed during the study (2010–2015) included the following: CaminoPro Pudding with Glytactin, Glytactin Bettermilk, Glytactin COMPLETE 15, Glytactin RESTORE, Glytactin RESTORE LITE, Glytactin RTD 15, and Glytactin SWIRL Caramel.

e Subjects classified with classical PKU have a genotype and lack of response to sapropterin dihydrochloride that are consistent with a severe PKU phenotype. Subjects classified with variant PKU have a phenylalanine hydroxylase genotype and/or response to sapropterin dihydrochloride that was consistent with a milder PKU phenotype. Mutation names are defined at http://www.pahdb.mcgill.ca and http://www.biopku.org.

f Kruskal-Wallis test was used.

**Table 2:** Group performance on Delis-Kaplan Executive Function System in subjects with phenylketonuria.

	**AA-MF**	**GMP-MF**	
**Delis-Kaplan Executive Function**	**Mean±SD**	**Mean±SD**	***p*-value**
**System categories**			
**Verbal fluency**			
Letter fluency scaled	9.40±3.67	9.00±3.01	0.3462
Category fluency scaled	10.90±3.12	10.23±3.17	0.2231
Category switching scaled	9.66±2.68	9.63±3.59	0.7373
Category switching: total switching accuracy	10.45±2.44	10.10±3.14	0.3953
Letter fluency vs category fluency scaled	8.57±2.97	8.77±3.86	0.7486
Category switching vs category fluency scaled	8.97±3.29	9.43±3.70	0.4538
Category switching percent switching accuracy scaled	11.24±1.94	10.97±2.51	0.5118
**Design fluency**			
Filled dots	9.62±3.26	9.80±2.91	0.5548
Empty dots only	9.93±3.13	9.53±3.26	0.2947
Switching	10.21±2.53	10.27±2.69	0.838
Design fluency total correct: composite	10.31±3.30	10.27±3.36	0.9544
Combined filled+empty dots: composite	10.00±3.14	9.97±2.87	0.8965
Switching vs combined filled contrast	10.21±2.43	10.30±2.12	0.9384
Percent design accuracy scaled score	8.76±3.04	9.37±2.57	0.2537

Values are mean±SD compared with normative sample performance as reflected in

scaled scores. Normative data, mean=10, SD=3, *n*=29–30.

AA-MF, amino acid medical foods; GMP-MF, glycomacropeptide medical foods.

**Table 3:** Group performance on Cambridge Neuropsychological Test Automated Battery in subjects with phenylketonuria.

	**AA-MF**	**GMP-MF**	
**Cambridge neuropsychological**	**Mean±SD**	**Mean±SD**	***p*-value**
**test automated battery categories**			
**Stockings of Cambridge**			
SOC mean initial thinking	-0.113±1.26	0.184±0.81	0.127
SOC mean subsequent thinking	0.563±0.57	0.078±1.29	0.196
SOC problems solved in minimum moves	0.050±0.99	-0.52±1.02	0.172
**Spatial span**			
SSP length	0.389±1.29	0.201±1.08	0.441
**Spatial working memory**			
SWM between errors	0.278±1.22	0.014±0.97	0.087
SWM strategy	0.458±1.50	0.080±1.12	0.086

Values are Z-scores mean±SD, *n*=22–24, Standard scores are

mean=0, SD=1. Higher Z scores=better performance.

AA-MF, amino acid medical foods; GMP-MF, glycomacropeptide medical foods.

## 2 Experimental design

**Fig. 1:**
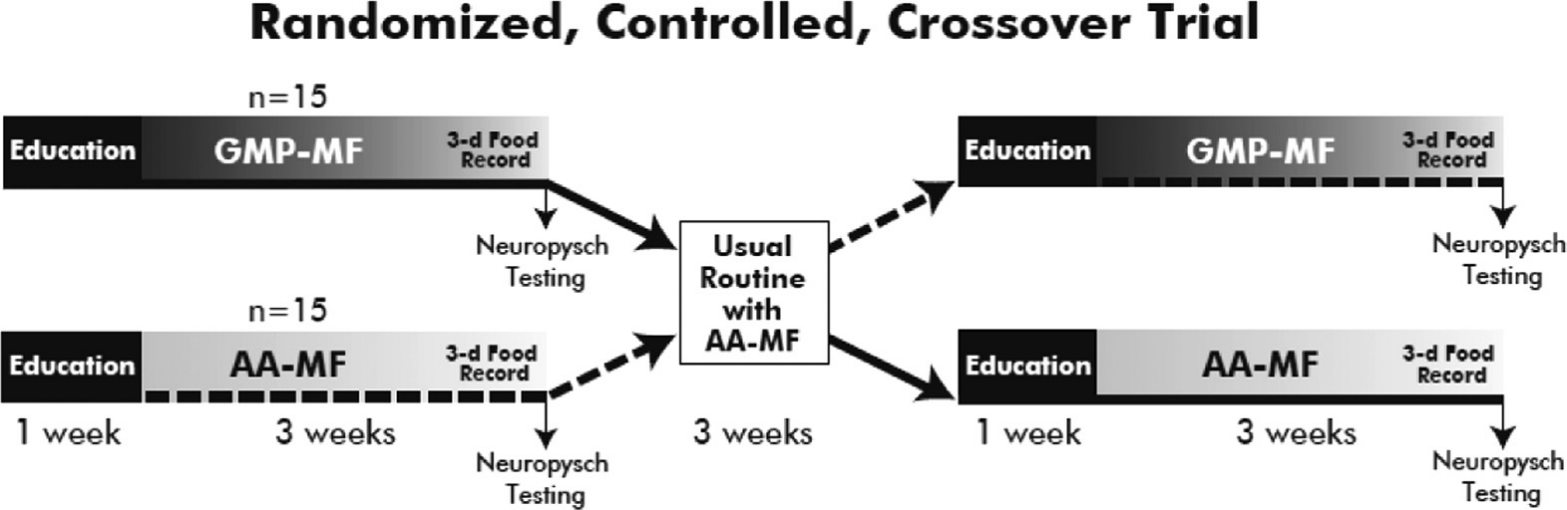
Experimental design.

Thirty subjects with early-treated phenylketonuria, 20 with classical and 10 with a milder or variant form of phenylketonuria, completed the clinical trial [1]. The experimental design was a 2-stage, randomized, controlled, crossover trial where subjects followed their usual low-phenylalanine diet in combination with amino acid medical foods and glycomacropeptide medical foods for 3-weeks each at home (Fig. 1). The protocol included: a one-week baseline period for diet education and orientation to the protocol while consuming the usual amino acid medical foods, the first 3-week dietary treatment with glycomacropeptide medical foods or amino acid medical foods, a 3-week washout period with return to the usual amino acid medical foods, a 1-week baseline period, and lastly the second 3-week dietary treatment with the glycomacropeptide medical foods or amino acid medical foods.

## 3 Materials and methods

Daily amino acid intake from the whole diet, medical foods, and natural food was calculated from consecutive 3-day food records at the end of the 3-week amino acid medical foods and glycomacropeptide medical foods treatments (Table 1). Amino acid calculations were performed by a Registered Dietitian skilled in standardized diet entry using Food Processor SQL (ESHA, version 10.12.0).

Neuropsychological testing to assess executive function, the Delis-Kaplan Executive Function System™ (Pearson Canada Assessment, Inc. Ontario, Canada), and the Cambridge Neuropsychological Test Automated Battery (Cambridge Cognition Ltd, Cambridge, UK) was conducted by research staff trained in the administration of standard psychological assessments under the supervision of a licensed psychologist after following the amino acid medical foods and glycomacropeptide medical foods treatments for 3 weeks (Table 2, Table 3).

## Funding

This work was supported by Department of Health and Human Services Grants R01 FD003711 from the FDA Office of Orphan Products Development to Ney, P30-HD-03352, T32 DK007665 to Ney, and by the Clinical and Translational Science Award (CTSA) program, through the NIH National Center for Advancing Translational Sciences (NCATS), Grant UL1TR000427. Cambrooke Therapeutics, Inc. donated the glycomacropeptide medical foods used in this study.
